# Effects of cataracts on flicker electroretinograms recorded with RETeval™ system: new mydriasis-free ERG device

**DOI:** 10.1186/s12886-016-0200-x

**Published:** 2016-03-05

**Authors:** Gen Miura, Yosuke Nakamura, Eiju Sato, Shuichi Yamamoto

**Affiliations:** Department of Ophthalmology and Visual Science, Chiba University Graduate School of Medicine, Inohana 1-8-1, Chuo-ku, Chiba, 260-8670 Japan

**Keywords:** Electroretinogram (ERG), Flicker ERG, Cataract, RETeval

## Abstract

**Background:**

The purpose of this study was to evaluate the effects of cataracts on the flicker electroretinograms (ERGs) recorded with the RETeval™ system under mydriatic-free conditions.

**Methods:**

This was a retrospective study of 82 eyes of 60 patients with cataracts and 52 eyes of 38 patients who were pseudophakic. Flicker ERGs were recorded with the RETeval™ system (LKC Technologies, Gaithersburg, MD) under mydriatic-free condition with skin electrodes. Flicker ERGs were elicited by white light delivered at a frequency of 28.3 Hz and intensity of 8 Td-s. The implicit times and amplitudes of the ERGs recorded from the Grade 2 cataract, Grade 3 cataract, and pseudophakic groups were compared.

**Results:**

The mean amplitude was significantly smaller in both cataract groups than the pseudophakic group (Grade 2 cataract vs pseudophakic group, *P* < 0.0001; Grade 3 cataract vs pseudophakic group, *P* < 0.0001; Grade 2 cataract vs Grade 3 cataract, *P* = 0.027).

The mean implicit times was significantly longer in both cataract groups than the pseudophakic group (Grade 2 cataract vs pseudophakic group, *P* = 0.046; Grade 3 cataract vs pseudophakic group, *P* = 0.0004; Grade 2 cataract vs Grade 3 cataract, *P* = 0.0084).

**Conclusions:**

The results indicate that the presence of Grade 2 or more cataracts will affect both the amplitude and the implicit time of the flicker ERGs. The presence of cataracts should be taken into consideration when interpreting the flicker ERG recorded with RETeval™.

## Background

The RETeval™ system (LKC Technologies, Gaithersburg, MD) is a noninvasive, mydriatic-free flicker electroretinogram (ERG) system that can elicit and record flicker ERGs. It uses skin electrodes and can be used on children, older patients, and bedridden patients that have difficulty maintaining a dorsal position for conventional ERG recordings. In addition, patients who have difficulty with mydriasis or with cornea electrodes should be suitable patients for the RETeval™ system. Because the flicker ERGs originate mainly from postreceptoral neural activity when the retina is stimulated with fast frequency flicker [1], the RETeval™ system can be used to assess these neural elements as well as detecting retinal ischemic changes in eyes with diabetic retinopathy and central retinal vein occlusion. Its ease of use and rapid recording times should make the RETeval™ system also valuable for evaluating the effectiveness of medical treatments [2–4].

A cataract is one of the most frequent ophthalmologic disease especially in the elderly, however its presence makes it difficult to examine the normality of the retina. This is important because it would not be practical for patients to undergo cataract surgery if the retina is dysfunctional. In addition, many of the patients with diabetic retinopathy and retinal vein occlusion are elderly and have cataracts.

The RETeval™ system should be a practical method to assess the retinal function in these eyes with a cataract because it can be performed rapidly, about 1 min/eye, without mydriasis and topical anesthetics. Therefore, it is important to determine how cataracts would affect the results of RETeval™ system. There have been studies that examined the relationship between the conventional ERGs and cataracts [5, 6], but as best we know, there has not been any studies published on the effects of cataracts on the flicker ERGs recorded with the RETeval™ system of a mydriatic-free condition.

Thus, the purpose of this study was to evaluate the effects of cataracts on the flicker ERGs recorded with the RETeval™ system. To accomplish this, we recorded flicker ERGs with the RETeval™ system from patients with different degrees of cataract and patients who were pseudophakic. We compared the amplitudes and implicit times of the flicker ERGs recorded from the three groups.

## Methods

This was a retrospective study of 82 eyes of 60 patients with cataracts and 52 eyes of 38 patients who were pseudophakic, i.e., who had an implanted intra ocular lens (IOL). All of the pseudophakic patients had undergone cataract surgery at the Chiba University Hospital. The procedures used in this study conformed to the tenets of the Declaration of Helsinki and were approved by the Institutional Review Board of Chiba University Hospital (number 2075). An informed consent was obtained from each patient for the surgery and for the ERG recordings.

All patients had a complete ophthalmic examination including measurements of the best-corrected visual acuity (BCVA), intraocular pressure, slit-lamp examination, and indirect ophthalmoscopy. The BCVA was measured monocularly using a Japanese standard Landolt ring chart (System Charts SC-2000 Nidek Instruments, Gamagori, Japan) at a test distance of 5 m. The decimal visual acuity values were converted to the logarithm of the minimum angle of resolution (logMAR) units for the statistical analyses.

The subjects with cataracts were divided into Grade 2 and Grade 3 cataract groups based on the Emery-Little classification [7].

Subjects with an implanted intraocular lens (IOL) were placed in the pseudophakic group and tested in the same way. Thus, the implicit times and amplitudes of the ERGs of the Grade 2 cataract, Grade 3 cataract, and pseudophakic group were compared.

There were 82 eyes of 60 patients (29 men and 31 women) whose mean age was 76.2 ± 6.4 years with a range of 62–87 years in the two cataract groups. The patients did not have any other abnormalities in the anterior segment, media, and fundus and were divided into Grade 2 or Grade 3 cataract groups. There were 60 eyes of 43 patients in the Grade 2 cataract group and22 eyes of 17 patients in the Grade 3 cataract group. The mean age in the Grade 2 group was 75.2 ± 6.2 years (range, 64–87 years) and that in the Grade 3 group was 78.5 ± 6.2 years (range, 67–87 years).

We evaluated 52 eyes of 38 patients (19 men and 19 women) in the pseudophakic group. The mean age was 75.7 ± 5.6 years (range, 63–87 years). All eyes had undergone phacoemulsification and intraocular lens implantation surgery through a superior sclera-corneal incision between June 2014 and July 2015 without any complications. Yellow-colored acrylic foldable intraocular lenses were implanted in all cases. Cases without any surgery-related complications and cases without any abnormalities in the anterior segment or fundus before and after cataract surgery were included for pseudophakic group. Eyes with refractive errors (spherical equivalents) greater than 6 diopters, and patients diagnosed with diabetes mellitus were excluded from this study.

Flicker ERGs were recorded with the RETeval™ system (LKC Technologies, Gaithersburg, MD) from all patients under mydriatic-free conditions. The pupil sizes of all subjects were in the range of 2–4 mm during ERG recording. Skin electrodes were used to pick up the ERGs that were elicited by white light at a frequency of 28.3 Hz and intensity of 8 Td-s, which is the recommended default setting for flicker ERGs for eyes without dilation in the RETeval™ system. No background light was used in this study. The contralateral eye was covered during examination. We used the values of amplitudes and implicit times of the fundamental component that were automatically analyzed, reconstructed and displayed by the RETeval™ system using a special algorithm.

Wilcoxon signed ranks test and Bonferroni multiple comparison tests were used to determine the significance of the differences in the amplitudes and implicit times of the flicker ERGs for the Grade 2, Grade 3, and pseudophakic groups. Pearson correlations tests were calculated to determine the significance of the correlations between the age and the amplitudes and implicit times of the flicker ERGs. A *P* value of <0.05 was considered statistically significant.

## Results

The mean BCVA was 0.26 ± 0.24 logMAR units with a range of 1.10 to −0.08 logMAR units for the Grade 2 cataract group, 0.67 ± 0.51 logMAR units with a range of 2 to 0.15 logMAR units for the Grade 3 cataract group, and −0.01 ± 0.12 logMAR units with a range of 0.52 to −0.08 logMAR units for the pseudophakic group. The BCVA was significantly reduced by the cataracts (Grades 2 cataract vs pseudophakic group, *P* < 0.0001; Grade 3 cataract vs pseudophakic group, *P* < 0.0001; Grade 2 cataract vs Grade 3 cataract, *P* < 0.0001).

The average amplitude of the flicker ERGs was 9.2 ± 3.7 μV for the Grade 2 cataract group, 7.1 ± 2.1 μV for the Grade 3 cataract group, and 13.0 ± 4.4 μV for the pseudophakic group. The average implicit time was 35.5 ± 1.8 ms for the Grade 2 cataract group, 37.0 ± 2.1 ms for the Grade 3 cataract group, and 34.7 ± 1.8 ms for the pseudophakic group.

**Fig. 1:**
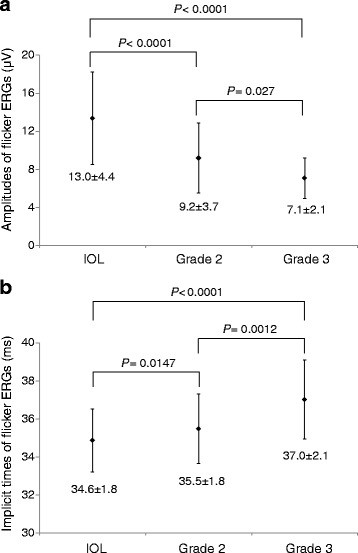
Amplitudes and implicit times of the flicker ERGs recorded with the RETeval™ system. Error bars are the standard deviations. **a**: Amplitudes of the flicker ERGs in the pseudophakic group, Grade 2 cataract group and Grade 3 cataract group. Amplitudes are significantly decreased in the cataract groups. **b**: The implicit times of the flicker ERGs of the pseudophakic group, Grade 2 cataract group, and Grade 3 cataract group. Implicit times are significantly prolonged in the cataract groups. Implicit times also show the significant difference between Grade 2 cataract group and Grade 3 cataract group

The mean amplitude of the flicker ERGs was significantly smaller in both cataract groups than the pseudophakic group (Grade 2 cataract vs pseudophakic group, *P* < 0.0001; Grade 3 cataract vs pseudophakic group, *P* < 0.0001; Grade 2 cataract vs Grade 3 cataract, *P* = 0.027; Fig. 1a).

The mean implicit times was significantly longer in both cataract groups than the pseudophakic group (Grade 2 cataract vs pseudophakic group, *P* = 0.0147; Grade 3 cataract vs pseudophakic group, *P* < 0.0001). The mean implicit times of the flicker ERGs in the Grade 2 cataract was significantly shorter than that in the Grade 3 cataract group (*P* = 0.0012; Fig. 1b).

**Fig. 2:**
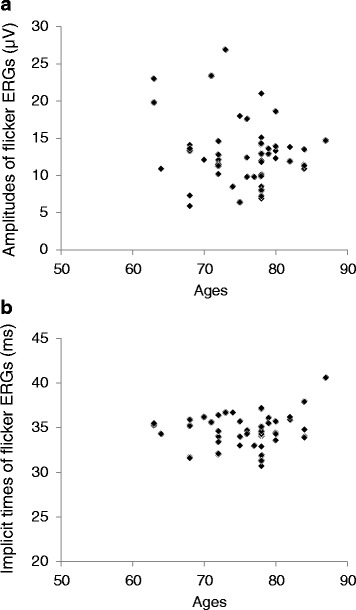
Correlations between the flicker ERGs and ages. **a**: The amplitudes of the flicker ERGs of all three groups recorded with the RETeval™ system were not correlated with the ages (*r* = −0.078, *P* = 0.59, *n* = 52; Pearson correlation coefficient). **b**: The implicit times of the flicker ERGs of all three groups were also not correlated with the ages (*r* = 0.095, *P* = 0.51, *n* = 52; Pearson correlation coefficient)

The age of IOL group did not show significant correlation between both the amplitudes (r = −0.078, *P* = 0.59; Fig. 2a) and the implicit times (*r* = 0.095, *P* = 0.51; Fig. 2b) of flicker ERGs with the RETeval™ system.

In the two cataract groups, 70 eyes had nuclear cataract and 12 eyes had cortical cataract.

The average amplitude of the flicker ERGs was 8.7 ± 3.5 μV for eyes with nuclear cataract and 8.5 ± 3.2 μV for eyes with cortical cataract. There was no significant difference between amplitudes of both types of cataract (*P* = 0.29).

The average implicit time was 36.0 ± 1.9 ms for eyes with nuclear cataract and 35.3 ± 2.4 ms for eyes with cortical cataract. There was no significant difference between implicit times of both types of cataract (*P* = 0.64).

## Discussion

Some of the earlier studies reported that the amplitude of flash ERGs recorded with conventional systems were significantly reduced in eyes with a cataract but other studies reported that they were not reduced [8, 9]. Although a cataract can reduce the intensity of the stimulating light which would reduce the amplitude of the ERGs, it also scatters the light thus stimulating a larger area of the retina which would increase the amplitude of the ERGs [10]. Previous studies had shown that a cataract will reduce the density of the multifocal ERGs (mfERGs) in the macular area but increase the peripheral mfERGs because of the light scattering [11, 12]. These differences in the stimulating conditions caused by cataracts may explain the differences in the amplitudes reported.

The flicker ERGs recorded with the RETeval™ system were done under mydriatic-free conditions, and the pupils would be constricted with the continuous stimulation. Thus, it was assumed that light scattering might be reduced which should then reduce the amplitude of the flicker ERGs. In addition, the contribution of the peripheral retina to the flicker ERGs should be minimal because the flicker ERGs originate mainly from the cone system.

Recently Kato et al. reported that as pupil size increased, the implicit times of the flicker ERGs recorded with RETeval™ system were significantly prolonged for larger pupil sizes. They assumed that the Stiles-Crawford effect is explainable reason for their results in that paper [13]. However, they showed that there were no significant differences in implicit times under 6 mm pupil size in their report.

As best we know, there are no reports on the relationship between the implicit times of the flicker ERGs and media opacities. In our patients, the average implicit time of the Grade 3 cataract group was significantly longer than that of eyes in the Grade 2 cataract group and the pseudophakic group. Thus, we conclude that the implicit times of the flicker ERGs were significantly affected by cataracts as with the amplitudes.

It has been reported that the amplitudes of the conventional ERGs and the mfERGs were affected by the age [14, 15]. In general, the b-wave amplitudes of conventional flash ERGs and the density of the mfERGs decreased with increasing age. However, it is possible that these results might have been affected by cataracts.

In our study, we showed that the age of IOL group was not significantly correlated with both the amplitudes and the implicit times of flicker ERGs with the RETeval™ system.

These results indicated that flicker ERGs elicited by the stimulus described above with the RETeval™ system under mydriatic-free conditions are not affected by ages.

In addition, some limitations of the current study should be noted. One limitation was the setting of RETeval™ system. We used intensity of 8 Td-s, which is the recommended default setting for flicker ERGs for eyes without dilation in the RETeval™ system. However, this stimulus was much weaker than that for light-adopted 3.0 flicker ERG which is recommended by International Society for Clinical Electrophysiology of Vision [16]. These settings of the light intensities might be the cause of the variations of the results. Therefore, it was assumed that the differences in the amplitudes and implicit times might decrease if the ERGs were elicited from stronger light stimuli in the three groups.

Another limitation of this study was that we compared eyes of different patients. Individual differences of the values of ERG might also affect the results. Further investigations with various pupil sizes, various light intensities, patients with more severe cataract than grade 3 cataract or the same individual eye before and after cataract surgery are needed.

RETeval™ system needs to measure the pupil size to keep a constant flash retinal illuminance. There were no failures to record flicker ERGs with RETeval™ system in all examination of this study period except few cases that corneal opacities prevented a proper detection of the pupil. This fact indicated that RETeval™ system is the simple and easy to examine unless the system could not detect pupil properly.

## Conclusion

In conclusion, the presence of Grade 2 or more dense cataracts may affect both of the amplitudes and the implicit times of the flicker ERGs, and it should be taken into consideration when interpreting the flicker ERG recorded with the RETeval™ system.

## References

[1] Kondo M, Sieving PA (2001). Primate photopic sine-wave flicker ERG: vector modeling analysis of component origins using glutamate analogs. Invest Ophthalmol Vis Sci.

[2] Holm K, Schroeder M, Lövestam AM. Peripheral retinal function assessed with 30-Hz flicker seems to improve after treatment with Lucentis in patients with diabetic macular oedema. Doc Ophthalmol. 2015; in press 10.1007/s10633-015-9495-9 25773362

[3] Kjeka O, Bredrup C, Krohn J (2007). Photopic 30 Hz flicker electroretinography predicts ocular neovascularization in central retinal vein occlusion. Acta Ophthalmol Scand.

[4] Larsson J, Bauer B, Andre’asson S (2008). The 30-Hz flicker cone ERG for monitoring the early course of central retinal vein occlusion. Doc Ophthalmol.

[5] Galloway NR (1988). Electrophysiological testing of eyes with opaque media. Eye.

[6] Tam WK, Chan H, Brown B (2004). Effects of different degrees of cataract on the multifocal electroretinogram. Eye.

[7] Emery JM, Little JH (1979). Surgical Techniques, Complications and Results. Phacoemulsification and Aspiration of Cataract.

[8] Cruz RD, Adachi-Usami E (1989). Quantitative evaluation of electroretinogram before cataract surgery. Jpn J Ophthalmol.

[9] Hurst MA, Douthwaite WA (1993). Assessing vision behind cataract—a review of methods. Optom Vis Sci.

[10] de Waard PW JKIJ, van den Berg TJ, de Jong PT (1992). Intraocular light scattering in age-related cataracts. Invest Ophthalmol Vis Sci.

[11] Chan HL, Siu AW, Yap MK, Brown B (2002). The effect of light scattering on multifocal electroretinography. Ophthalmic Physiol Opt.

[12] Tam A, Chan H, Brown B, Yap M (2004). The effects of forward light scattering on the multifocal electroretinogram. Curr Eye Res.

[13] Kato K, Kondo M, Sugimoto M (2015). Effect of Pupil Size on Flicker ERGs Recorded With RETeval System: New Mydriasis-Free Full-Field ERG System. Invest Ophthalmol Vis Sci.

[14] Karpe G, Rickenbach K, Thomasson S (1950). The clinical electroretinogram. I. The normal electroretinogram above fifty years of age. Acta Ophthalmol.

[15] Birch DG, Anderson JL (1992). Standardized full- field electroretinography. Normal values and their variation with age. Arch Ophthalmol.

[16] McCulloch DL, Marmor MF, Brigell MG (2015). ISCEV standard for full-field clinical electroretinography (2015 update). Doc Ophthalmol.

